# New Specific Kinesthetic Differentiation Tests for Female Volleyball Players: Reliability, Discriminative Ability, and Usefulness

**DOI:** 10.3390/jfmk8020063

**Published:** 2023-05-12

**Authors:** Karla Đolo, Zoran Grgantov, Goran Kuvačić

**Affiliations:** Faculty of Kinesiology, University of Split, 21000 Split, Croatia; karla.djolo@gmail.com (K.Đ.); zoran.grgantov@kifst.eu (Z.G.)

**Keywords:** coordination abilities, inter-positional difference, volleyball techniques

## Abstract

This study aimed to determine the test-retest reliability and discriminative ability of five sport-specific kinesthetic differentiation ability tests in female volleyball players. The sample of participants consisted of 98 female volleyball players aged 15.20 ± 1 years from six clubs in Bosnia and Herzegovina. Kinesthetic differentiation ability was determined by the overhead passing test, forearm passing test, float service with a net test, float service without a net test, and float service 6 m from the net test. To estimate test-retest reliability, a sub-sample of 13 players performed all tests on two testing occasions. Furthermore, the discriminative ability of the tests was determined by analyzing the performance between players of different playing positions and situational performances. Parameters of the intraclass correlation coefficient (ICC) were excellent (0.87–0.78) in all tests except for the float service with the net test, whose reliability was good (0.66). For the absolute reliability estimates, the SEM was higher than SWC (0.2) for all variables except the float service 6 m from the net test, and the SEM was lower than SWC (0.6, 1.2). One-way ANOVA detected no statistically significant inter-positional differences in all five tests (*p* > 0.05). A significant difference was found between less and more successful players (*p* < 0.01) for all applied tests. The results of this study show that a specific battery test is a reliable and valid measure and can be used to monitor kinesthetic differentiation ability in young female volleyball players.

## 1. Introduction

Modern volleyball is a dynamic sport that involves multidirectional accelerations from landing to jumping, sudden starts and stops, changes of direction, attack, and defensive actions that require high levels of technical and tactical skills [1,2,3]. To achieve all these skills at a young age, training must be based on coordination abilities to excel in all game segments [4]. Otherwise, it is unlikely for athletes to reach their full potential later in life. Most studies emphasize the importance of sensitive phases as critical periods of enhanced children’s coordination development [5,6]. In contrast, others doubt the existence of these phases and conclude that motor learning capacity can also improve in adolescence and later [7,8,9]. Nowadays, all researchers must keep in mind that a high level of skills cannot be possible if a child has not reached neural maturity [10].

Previous research highlights the significance of improving technical and tactical skills by applying all five basic coordination abilities: kinesthetic differentiation, spatial orientation, rhythm, balance, and reaction abilities [11,12]. Moreover, supplementing general coordination abilities with specific coordination will facilitate the learning process [13,14,15]. A competitive sport such as volleyball is challenging to master due to many technical aspects that require accuracy, such as serving, passing, and setting [16]. All of them represent the so-called ball feeling, partner feeling, and opponent feeling [17], which we can link with the most important coordination ability—kinesthetic differentiation. Researchers define it as a sense of upper and lower extremities and body movement where kinesthetic memory helps players to remember their motor movements, such as the skill needed for receiving service from different angles [7,18,19]. To successfully master this ability, players need to go through a large number of repetitions, monitored constantly until they no longer need to think about the movement; they just “do it” [20].

Until recently, many researchers have focused on constructing and developing tests to assess volleyball’s general kinesthetic differentiation of lower and upper limbs [21,22,23,24]. General coordination tests, such as kinesthetic differentiation, are suitable for children before playing volleyball or for beginner volleyball players. They cannot perform specific tests because they have not yet perfected volleyball techniques. Therefore, it is common to apply general tests for jumping, throwing, and similar skills for players up to 14 years old. However, children aged 14 to 15 have already mastered volleyball techniques enough to be able to use specific coordination tests.

Recent studies have noted the need for sport-specific tests, which have contributed to modern sports and are more appropriate than standard tests because they are similar to real sports situations [25]. Nevertheless, there are insufficient reports on specific kinesthetic differentiation ability, especially in volleyball [1,26,27]. In short, these researchers focused on measuring skills to identify talented volleyball players by applying specific tests.

Specific volleyball coordination tests can be used as a means of talent identification to monitor the progress of volleyball players over a certain period. We can assume that more talented players will adopt volleyball techniques more rapidly, progress faster over time, and reach a higher level of skill [28]. Another possibility is to use them as a tool to assess the current level of volleyball technique. Therefore, the study aimed to examine the test-retest reliability and discriminative ability of new sport-specific kinesthetic differentiation ability tests in female volleyball players.

## 2. Materials and Methods

### 2.1. Study Design

This study had two stages: (1) test-retest reliability and (2) discriminative ability assessment. To assess the test-retest reliability, a subgroup of 13 participants underwent the same tests on two separate occasions, with a seven-day interval between them. The discriminative ability of the tests was determined by analyzing the group differences among players of different playing positions and situational performances.

During the investigation days, the players were instructed to avoid any factors that could hinder their effort during testing. This included refraining from consuming caffeine-containing beverages and low-fiber diets 24 h before and during the investigation in order to minimize any interference with the testing. Additionally, the athletes were instructed to refrain from engaging in physical activity 48 h before and during the investigation. To prevent circadian variations, all tests were conducted in the morning, starting at 8 am [29].

The testing was conducted in June 2021 with an ambient temperature ranging from 21 to 24 °C. At the beginning of the testing session, a standardized warm-up consisting of 10 min of jogging and mobility exercises was conducted. Players performed three maximum trials, followed by six trials of each skill. All tests were demonstrated and conducted by professionals, members of the Faculty of Kinesiology, University of Split, who introduced them to all testing procedures. Participants were given a briefing on the testing procedures prior to beginning any of the tests.

### 2.2. Participants

The study was conducted on 98 young female volleyball players, aged 15.20 ± 1.00 years, from six clubs in Bosnia and Herzegovina. The players specified their playing positions, which included setter (*n* = 17), passer-hitter (*n* = 35), opposite hitter (*n* = 16), middle blocker (*n* = 19), and libero (*n* = 11). Coaches from six volleyball clubs agreed to participate and received a clear explanation of the study. All players had been involved in at least two years of training. Informed consent was obtained from parents before players were permitted to participate. The inclusion criteria for players were being at a stage of specialized basic training with no musculoskeletal or psychophysical disorder. A professional volleyball coach supervised and regulated young athletes’ training programs, which mainly comprised technical and tactical volleyball training and some physical conditioning sessions. On average, athletes trained for approximately 2 h per session, four days per week.

The situational performance of the players was assessed by combining 2 criteria [1]: (1) Placement of teams in the competition (Super League and 1st League); (2) Quality of individual players within their team where the coaches categorize their team players into 3 groups: group 1 including leading team players; group 2 including the rest of starting players and players entering the game, thus contributing to team result; and group 3 including players who very rarely or never enter the game. As presented in Table 1, a combination of these assessment criteria, each player is scored 1–5. More successful players were those that scored 5 and 4, and less successful were the ones that scored 3, 2, and 1. Therefore, the sample was divided into two subsamples: less successful (*n* = 59) and more successful (*n* = 39) players.

During the experiment, eight players who did not have an assigned playing position were excluded. Additionally, three players were excluded because they could not perform the service tests.

### 2.3. Experimental Procedures

The kinesthetic differentiation ability was assessed using five different volleyball skill tests: forearm passing, overhead passing, and three different standing float service tests, all on an indoor floor surface.

#### 2.3.1. Forearm Passing Test (FPT)

**Figure 1:**
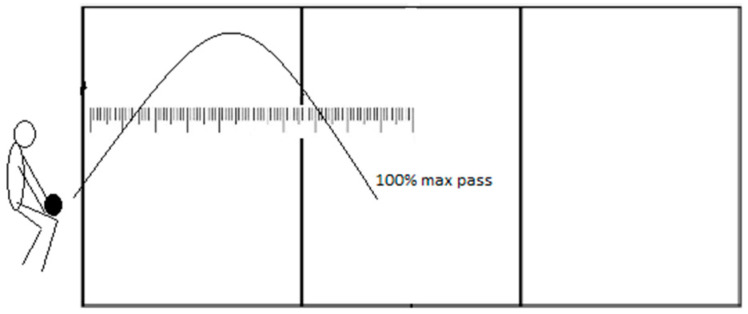

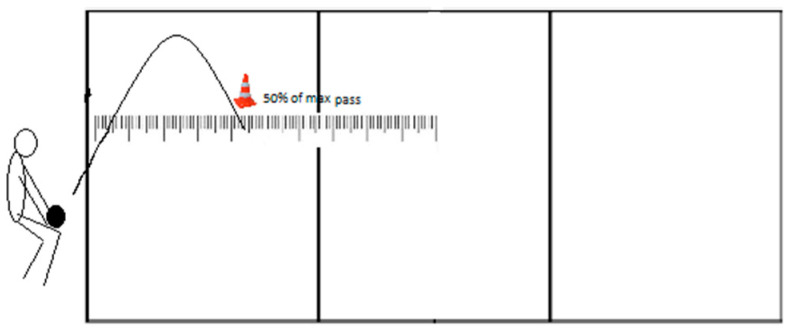
Visual representation of forearm passing test.

In the first task, the athlete stands in front of a line with a ball in their hands (Figure 1). Their task is to throw the ball in front of themselves and forearm pass the ball three times as far as possible. A measuring scale is placed on the floor surface, and the ball bounces along that measuring scale. The measurer counts 50% of the athlete’s maximum pass and marks that target with a cone. The kinesthetic differentiation during forearm passing is evaluated in the second part of the test, where athletes need to pass the ball as close to the cone as possible. The measurer records six attempts. Therefore, the test results represent the sum of deviations in centimeters in those six attempts (absolute values regardless of negative sign).

#### 2.3.2. Overhead Pass Test (OPT)

**Figure 2:**
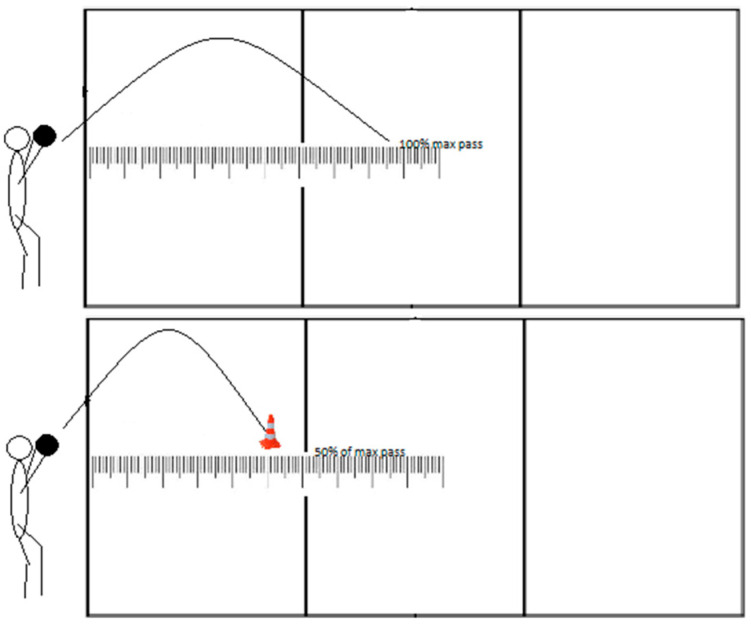
Visual representation of overhead passing test.

The athlete begins by holding the ball and throwing it overhead to themselves (Figure 2). They then play the ball with an overhead pass as far as possible three times. The measuring scale is also placed on the floor surface, and the ball bounces along that measuring scale. After the athlete completes the three maximum passes, the measurer counts 50% of the maximum distance and marks that place with a cone. The kinesthetic differentiation during overhead passing is evaluated by asking athletes to overhead pass the ball as close to the cone as possible. The measurer records six attempts, and the test results represent the sum of deviations in centimeters in those six attempts (absolute values regardless of negative sign).

#### 2.3.3. Float Service Tests (FST)

The standing float service accuracy was evaluated in three different tests: (1) float service with a net (FSTnet), (2) float service without a net (FSTx), and (3) float service 6 m from the net (FST6m). In all three tests, athletes first had to serve three maximum attempts on the court from a service position, followed by six attempts aiming at a target. Therefore, the test results represent the sum of deviations in centimeters in those six attempts (absolute values regardless of the negative sign).

**Figure 3:**
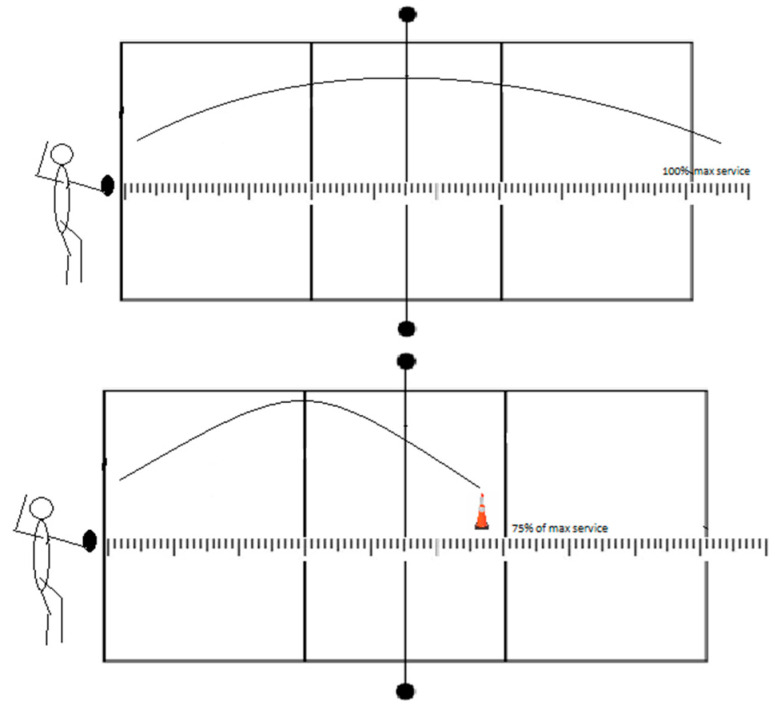
Visual representation of float service with a net test.

In the FSTnet, athletes stand behind the baseline while serving (9 m from the net; Figure 3). After three maximum services over the net, the measurer counts 75% of the athlete’s maximum serve and marks the target with a cone. Then, athletes have to serve the ball six times to that target. A measure of 75% was taken, unlike other tests, because the target would be farther from the net with this percentage. With 50%, the target for most participants would be near the net, and the test could not be performed since the net would interfere with hitting the target.

**Figure 4:**
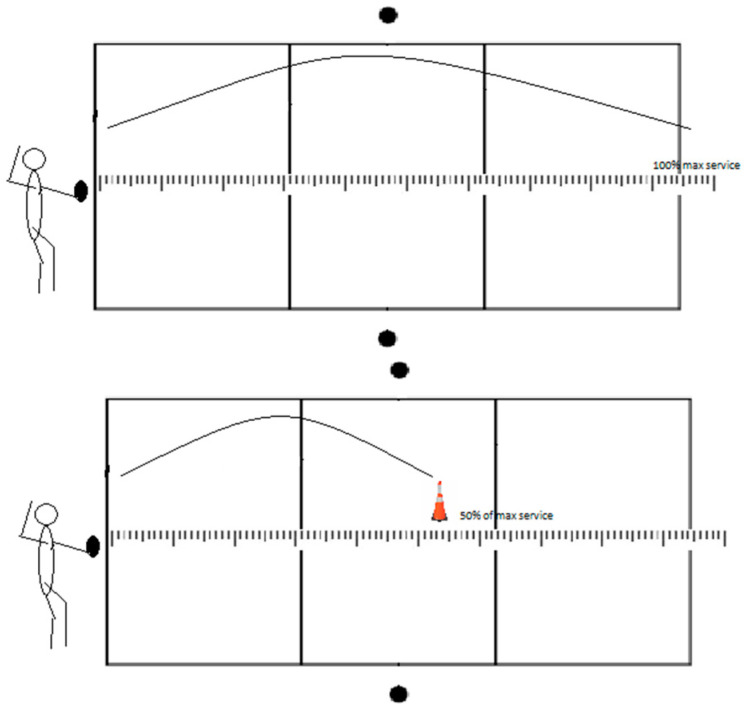
Visual representation of float service without a net test.

In the FSTx, the athlete serves in front of the baseline, imagining the net is on the court (Figure 4). The measurer counts 50% of the maximum serve and places the cone on that spot. Then, athletes have to serve the ball six times to that target.

**Figure 5:**
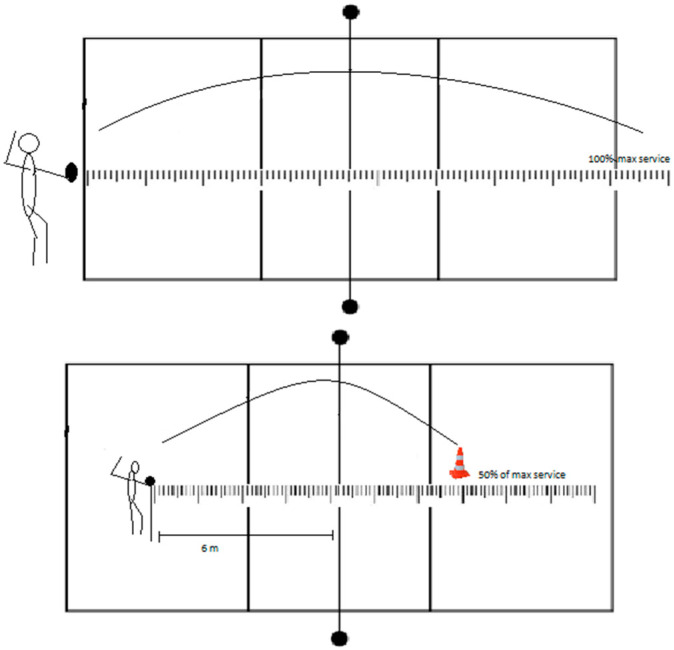
Visual representation of float service 6 m from the net test.

The third version of the test (FST6m) differs by positioning the athlete in front of the net after three maximum serves (Figure 5). The athlete serves three maximum serves from behind the baseline. Afterward, the athlete moves six meters from the net, from where they will serve the next six attempts. The measurer counts 50% of the maximum service and measures that distance, 6 m from the net. The athletes need to serve on the marked place over the net.

### 2.4. Statistical Analysis 

The intraclass correlation coefficient (ICC) was used on the subsample group with the same age to measure test-retest reliability. According to Koo and Li [30], the ICC was considered poor if less than 0.39, between 0.40 and 0.59 was fair, from 0.60 to 0.75 was good reliability, and considered excellent if larger than 0.75. Absolute reliability was analyzed to determine the usefulness of the specific kinesthetic differentiation tests by calculating the standard error of measurement (SEM) using the formula: [SEM = SD × √1 − ICC]. The smallest worthwhile change (SWC) was assumed by multiplying the between-participant standard deviation with different effect sizes (0.2, 0.6, and 1.2). The usefulness can be rated as “good”, “marginal”, and “satisfactory” when the SEM is below, similar, or higher than the SWC, respectively [31]. Furthermore, minimal detectable change (MDC) was analyzed with the following formula: [MDC = SEM × 1.96 × √2] to monitor progress so that intra-trial variations do not inaccurately suggest a change. The normality of the sample was tested using the Kolmogorov–Smirnov test (*p* > 0.05). The applied tests were assessed for their ability to distinguish differences between players in different playing positions and situational performance differences of young volleyball players in terms of discriminative ability. Therefore, one-way ANOVA was analyzed to determine differences in five positions (setter vs. passer-hitter vs. opposite player vs. middle blocker vs. libero), while the Student’s paired *t*-test was used to determine if there is a difference between situational performance (successful vs. less successful). Additionally, Cohen’s d was calculated with the magnitude of d was qualitatively interpreted using the following thresholds: <0.2, trivial; 0.2 to 0.6, small; 0.6 to 1.2, moderate; 1.2 to 2.0, large; and 2.0 to 4.0, very large [32]. The statistical analysis was performed using SPSS Statistics 27.0 for Windows. The statistical significance for all tests was set at *p* < 0.05.

## 3. Results

The relative and absolute reliability of specific kinesthetic differentiation ability tests is presented in Table 2. The values of ICC range from 0.66 to 0.87, indicating excellent test-retest reliability in all tests except in the FSTnet, whose reliability was rated as good. The SEM values for almost all of the test measurements were low except in the FSTnet. Furthermore, SEM values were higher than SWC for all variables except for FST6m when the smallest level (0.2 multiplied by between-participants SD) was considered.

In Table 3, inter-positional differences were presented by applying ANOVA on the whole sample of female volleyball players. The results showed that there were no significant differences in all five specific tests (*p* > 0.05).

Table 4 shows the difference between less successful and more successful female volleyball players. Based on Table 4, successful young female volleyball players achieved better than less successful players in all five-specific kinesthetic-differentiation tests (*p* < 0.01).

## 4. Discussion

This study aimed to analyze the reliability, discriminative ability, and usefulness of new specific kinesthetic differentiation battery tests in young female volleyball players. Almost all tests had excellent test-retest reliability, except the FSTnet, whose reliability was good. These results agree with the findings of [33], who tested junior volleyball players in spiking, setting, serving, and passing techniques. Furthermore, performances of service change through different age groups and levels, as well as the accuracy of trajectory, which is critical for effectiveness in young age groups. In such a way, the intention for using three different service tests was to observe which were most suitable for use in this age group. Observing the results of three service tests, it can be seen that the FSTnet has the lowest result in reliability (0.66). Primarily, the biomechanical process of serving must be on a higher level to achieve correct consecutive repetitions. For players to successfully perform the service from a long distance and to have an accuracy of performance, they should have developed coordination along the kinetic chain [34]. Therefore, the service movement starts with placing the foot on the floor during a step; thus, the reaction of the floor occurs, which passes through the entire body. Moreover, young players are limited with the strength of lower and upper muscles, which leads to greater oscillations in the trials of this test. Accordingly, FST6m is the most convenient for testing reliability in young female volleyball players. Neither does the long distance exists between the player and the net nor does strength come to such an extent. The net placed at a greater distance from the service point represents a psychological barrier that pushes players to apply greater force, which results in greater oscillations. Furthermore, when youth players are serving 9 m away from the net, the target is close to the net on the other side, requiring the ball to go over the net in a higher arc while also using considerable force. This is a challenging combination that could present a problem even for more experienced and technically skilled players.

Upon analyzing the usefulness of the tests, it can be observed that the amount of measurement error and noise in the tests ranges from 0.1 to 0.15 m (1% to 32%), indicating that below 10% only differentiate in FPT and FST6m. Other tests, apart from FST6m, whose usefulness was rated as “good,” had small performance changes SWC (0.2) and showed “satisfactory” usefulness. Moreover, moderate variations could be detected as the SEM values were lower than SWC (0.6, 1.2) for all tests indicating “good” usefulness. Therefore, all kinesthetic differentiation tests could detect a real change that exceeds 0.6 and 1.2 times the standard test deviation [31]. Additionally, by observing the MDC (the real changes in measured performance from test and retest), values varied from 16, 19, 41, 22, and 0.03 cm. High values of FSTnet (0.41) may raise concerns regarding the precision of the measure. Consequently, all these tests have small targets, which influence the accuracy and lead to higher performance variations [35]. Specifically, other interactions may also influence testing consistency every time players are tested, such as fatigue, stress, pressure, and concentration [36].

For this study, the intention was to establish how well-applied tests will discriminate against young athletes. Therefore, the present study analyzed the discriminative ability with two objectives: (i) to evaluate are differences between playing positions; (ii) to evaluate differences between more successful and less successful young volleyball players in specific kinesthetic-differentiation battery tests. No significant inter-positional differences were observed in all five tests. Volleyball is a late-specialization sport; therefore, individual player position specialization does not begin until the age of 14–15. The young volleyball players in this study have only begun the specialization process, so the position-specific training has not yet impacted the specific development of certain abilities and skills. It is important to master a high level of general coordination abilities afterward by entering the specialization of playing positions to master complex volleyball techniques later on [12,22,28,37]. In addition, volleyball is a skill-based sport where complex performance techniques, both with and without the ball, are characteristic of all player roles. Therefore, it can be assumed that a high level of kinesthetic differentiation is equally necessary for successful play in all player positions in volleyball. Consequently, a significant difference was observed between less successful and more successful volleyball players for all tests. The results are consistent with the conclusions drawn by Pion et al. [28], who highlighted that differences between elite and sub-elite levels of play in volleyball are influenced by motor coordination. Motor coordination could play a crucial role in differentiating those who reach an expert level in female volleyball from those who do not. As such, the results provide support for the notion that overall motor coordination may serve as a useful predictor of an athlete’s potential for advancement in skill-based sports such as volleyball. Moreover, successful talent-identified junior volleyball players can be distinguished from unsuccessful ones based on their skill test results, specifically passing and serving technique evaluations by coaches, but not based on their physical characteristics [38].

These results highlight the significant importance of kinesthetic-differentiation ability in volleyball. This ability consists of three components: force (tension) as the strength of the movement, spatial (space) as the angle of joints, and temporal (time) through movement speed, which all contribute to more efficient volleyball techniques [36,39,40]. However, due to its complexity in defining learning methods and the relatively short contact time with the ball, it is essential to focus more on the development of specific kinesthetic-differentiation abilities in young volleyball players [20]. The accuracy of overhead and forearm passing is important for receiving serves, defending the court (especially for precisely playing balls that the opposing team did not hit with a strong spike but played lightly—which is common in younger age groups), as well as for setting up for a spike. The precision of serves enables serving into empty spaces that opposing serve receivers did not cover or targeting players who are known for poor serve reception.

This study has some limitations. Firstly, the sample only consisted of female athletes; future studies should include male athletes. Additionally, the reliability of the tests was established in a non-fatigued state, so further investigations are needed to assess their effectiveness when athletes are fatigued. Although the sample size was not small, it should be even larger when examining differences between playing positions. Lastly, only discriminant ability was analyzed in this study, and future investigations should establish convergent validity by comparing the results with other tests considered the gold standard. 

## 5. Conclusions

The findings of this study confirm the importance of kinesthetic differentiation ability in volleyball and the need for its development in young athletes. The newly constructed tests of specific kinesthetic differentiation in this study are reliable. Due to their discriminative ability among performance levels, they can be used in the talent identification process and to assess progress in individual player positions in volleyball. Coaches should be aware of the importance of kinesthetic-differentiation ability in complex tasks such as service tests, overhead passing, and forearm passing and incorporate appropriate training methods from an early age. It can be assumed that the FSTx test is suitable for even younger volleyball players than those in this study group, while the FST6m test is best for this group, and it can be assumed that the FSTnet test will be the best option for a cadet, junior, and senior players (U17 and older age groups).

## Figures and Tables

**Table 1:** The situational performance of the players according to the two criteria.

Team Placement	Within Team Players’ Quality Evaluated by the Coach		
	Group 1	Group 2	Group 3
Super League	5	4	3
1st League	3	2	1

**Table 2:** Results of the reliability and usefulness of the coordination tests.

	Test	Re-Test	ICC	SEM	SWC (0.2, 0.6, 1.2)	MDC
FPT (cm)	62.6 ± 28.7	51.5 ± 16.8	0.84	0.06	0.03, 0.09, 0.19	0.16
OPT (cm)	24.2 ± 11.1	34.8 ± 21.1	0.78	0.07	0.03, 0.09, 0.18	0.19
FSTnet (cm)	111.8 ± 64.6	110.2 ± 46.7	0.66	0.15	0.08, 0.24, 0.48	0.41
FSTx (cm)	59.6 ± 14	54.2 ± 21.7	0.77	0.08	0.03, 0.09, 0.19	0.22
FST6m (cm)	61.4 ± 34.1	77.5 ± 21.6	0.87	0.01	0.04, 0.13, 0.27	0.03

Legend: All values are presented as mean ± SD; ICC = intraclass correlation coefficient; SEM = standard error of measurement; SWC = smallest worthwhile change; MDC = minimal detectable change.

**Table 3:** Inter-positional differences analyzed by one-way ANOVA for the specific kinesthetic differentiation tests on the total sample of 98 young female volleyball players.

Variables	Setter (*n* = 17)	Passer-Hitter (*n* = 35)	Opposite Player (*n* = 16)	Middle Blocker (*n* = 19)	Libero (*n* = 11)	F	*p*
FPT (cm)	72.3 ± 32.9	57.6 ± 29	53.5 ± 25.6	59.4 ± 31.6	48.2 ± 25.3	0.51	0.76
OPT (cm)	40.1 ± 12	38.7 ± 16.8	44 ± 14.2	45.1 ± 18.4	36.5 ± 16.6	0.72	0.6
FSTnet (cm)	124 ± 97.2	109.6 ± 60.4	91.7 ± 37.9	154.3 ± 80.4	109.6 ± 21.6	2.27	0.06
FSTx (cm)	65 ± 21.5	72.2 ± 22.6	75.3 ± 37.1	77.8 ± 38.8	75.9 ± 21.6	0.41	0.84
FST6m (cm)	125.1 ± 67.8	119.6 ± 83.3	106.4 ± 63.4	175.8 ± 87.1	110.2 ± 64.2	1.51	0.2

Legend: All values are presented as mean ± SD; F—f test; *p*—probability value.

**Table 4:** Differences analyzed by paired *t*-test for the specific kinesthetic differentiation between less successful and more successful young female volleyball players.

Variables	Less Successful (*n* = 59)	More Successful (*n* = 39)	*t*-Value	*p*	ES	
					d	95% CI
FPT (cm)	72 ± 31.5	50 ± 24.1	3.92	<0.01	0.76	0.34–1.18
OPT (cm)	51.5 ± 18.6	35.4 ± 11.4	4.80	<0.01	1	0.56–1.42
FSTnet (cm)	154.2 ± 79.2	82.6 ± 24.5	5.44	<0.01	1.13	0.69–1.55
FSTx (cm)	93.9 ± 28.7	58.9 ± 17.5	7.08	<0.01	1.41	0.95–1.84
FST6m (cm)	178.1 ± 74.3	87.9 ± 54.6	6.06	<0.01	1.34	0.89–1.78

Legend: All values are presented as mean ± SD; *t*—*t* test; *p*—probability value; ES—effect size; d—Cohen d; 95% CI—95% confidence interval.

## Data Availability

Data can be provided on reasonable request.
